# Asynchronous partial contact motion due to internal resonance in multiple degree-of-freedom rotordynamics

**DOI:** 10.1098/rspa.2016.0303

**Published:** 2016-08

**Authors:** A. D. Shaw, A. R. Champneys, M. I. Friswell

**Affiliations:** 1 College of Engineering, Swansea University, Bay Campus, Fabian Way, Swansea SA1 8EN, UK; 2 Department of Engineering Mathematics, University of Bristol, Merchant Venturers Building, Woodland Road, Clifton BS8 1UB, UK

**Keywords:** rotating machines, internal resonance, whirl, stator contact, bifurcation

## Abstract

Sudden onset of violent chattering or whirling rotor–stator contact motion in rotational machines can cause significant damage in many industrial applications. It is shown that internal resonance can lead to the onset of bouncing-type partial contact motion away from primary resonances. These partial contact limit cycles can involve any two modes of an arbitrarily high degree-of-freedom system, and can be seen as an extension of a synchronization condition previously reported for a single disc system. The synchronization formula predicts multiple drivespeeds, corresponding to different forms of mode-locked bouncing orbits. These results are backed up by a brute-force bifurcation analysis which reveals numerical existence of the corresponding family of bouncing orbits at supercritical drivespeeds, provided the damping is sufficiently low. The numerics reveal many overlapping families of solutions, which leads to significant multi-stability of the response at given drive speeds. Further, secondary bifurcations can also occur within each family, altering the nature of the response and ultimately leading to chaos. It is illustrated how stiffness and damping of the stator have a large effect on the number and nature of the partial contact solutions, illustrating the extreme sensitivity that would be observed in practice.

## 1. Introduction

The lateral vibrations of rotating machinery are an important issue in many engineered systems [1,2]. Understanding of rotating machinery in linear operating regimes is well established, with classical methods from linear algebra being applied to deduce the whirl speeds of rotational systems with any number of degrees of freedom. However, there are many outstanding problems concerning nonlinearity in rotating systems, and these are leading to new approaches in a variety of industrial applications. One novelty compared with non-rotating vibration problems is that matrices associated with rotordynamic systems are highly non-normal due to the presence of gyroscopic forces. The interplay between the non-normality and nonlinear effects can lead to counterintuitive dynamical consequences (e.g. [3,4]).

By their nature, rotors are susceptible to geometric nonlinearity [5,6] and can experience potentially catastrophic effects from a nonlinear viscous phenomenon known as oil whip [7–9]. Patel & Darpe [10] noted that cracks in rotors also induce nonlinear effects and explored these effects with bifurcation analysis. Other forms of nonlinearity can arise through squeeze-film damping effects [11] or through additional automatic dynamic balancer mechanisms [4,12,13]. However, the focus for the current work is nonlinearity due to the rotor making contact with an external stator.

Rotor–stator contact is an issue that affects a wide variety of applications, from turbomachinery [14] to drilling for mineral extraction [15] and is driving a large body of research [16]. Many rotating devices incorporate magnetic bearing systems [17,18], and these must be designed with consideration of the consequences of a touch-down event in response to a failure or disturbance [19]. Ehrich [20] has compiled examples of numerous contact phenomena witnessed in tests on turbomachinery, including period-doubling bifurcation routes to chaos, subharmonic resonance and other surprising effects such as bearing phenomenon that lead to a rotor slowly ‘switching’ between two amplitudes of vibration.

Contact nonlinearities are typically due to friction during contact and discontinuous stiffness effects, and the typical response behaviours are classified by Jacquet-Richardet *et al.* within three classifications; *forward whirl annular motion*, *backward whirl motion* and *rebounds* [14]. Forward whirl annular motion occurs synchronously; the shaft whirls in constant contact with the stator, at the same angular speed and direction as the shaft rotation. This generates heat on the contacting side of the shaft, and this results in a bending known as the Newkirk effect [1,21]. Backward whirl motion again occurs with constant contact between rotor and stator, but in these cases the friction is sufficient to cause the shaft to whirl in the opposite direction to the shaft rotation. In many cases, there is zero relative sliding between the two surfaces, in which case the whirling is typically very rapid, known as dry whirl or dry whip. Owing to its potentially destructive nature, the stability and onset of the dry whirl condition has been widely studied (e.g. [22–25]). In addition, if torsional deformation of the shaft is considered, constant contact response may also include a component of torsional vibration [26], perhaps including complex stick-slip phenomena [27–30].

Rebound motion consists of intermittent contact between rotor and stator, and while many names for forms of this motion are used in the literature (chatter, intermittent contact, rattle, bouncing), we will henceforth refer to these responses as exhibiting *partial contact*. Some of the earliest work on partial contact motion was summarized in the review by Ehrich [20]. Hallmarks of such motion can be seen in the observation of sum and difference combination resonances in the response of impacting rotors [31], and a case where a non-rigid stator was shown to whirl [32]. Neilson & Barr [33] constructed an experimental rig for rotor–stator contact, and showed that the resulting spectral output had synchronous content (i.e. at integer ratios to the drive speed frequency) as well as asynchronous *sidebands*. During the 1980s, researchers began to view these systems through the framework of nonlinear dynamical systems theory [34,35] and later numerical and experimental studies began to show evidence of period doubling phenomena and chaos. For example, Muzsynska & Goldman [36] numerically demonstrated bifurcations to period-*n* motion and transitions to chaos under variation of shaft speed, with good qualitative comparison to experimental results. Chu & Zhang [37] used Floquet analysis of orbits of a contacting Jeffcott rotor to trace the progression to chaos; their results bore similarity to those of Muzsynska & Goldman [36], where brief regions of chaos were interspersed between wider regions of simpler period-*n* response. Edwards *et al.* [38] produced a numerical study of a single contacting disc on a shaft that showed chaotic responses at every integer and half-integer multiples of the first critical drive speed, and demonstrated that this behaviour could be significantly influenced by torsional effects.

In other work, Karpenko *et al.* [39] developed a piecewise smooth model for rotors experiencing frictionless impacts with a snubber ring, and in later work examined the response space of this system with bifurcation analysis, and revealed the basins of attraction for various solutions [40]. Pavlovskaia considered a similar system, but where the snubber ring springs were preloaded to create additional nonlinearity [41], and Karpenko *et al.* [42] went on to compare these predictions with experiment, where the experimental stator could rotate on a bearing to approximate the assumption of zero friction contact. Chu & Lu [43] studied a two- disc experimental system, showing some highly complex multi-period and quasi-periodic behaviour, although the authors comment that they could not directly trace the route into chaos due to experimental control issues. Further experimental data have been provided by Torkhani *et al.* [44] on a two disc experimental rig, which also demonstrated agreement with a numerical model.

Some researchers have employed harmonic balance methods to investigate these systems, instead of the piecewise-smooth methodology used in some of the works above. For example, Kim & Noah [45] found solutions using a method that combined harmonic balance with an alternating frequency time-domain analysis, that assumed two fundamental frequencies, to handle the observed effect that some response frequencies were not periodic in the drive speed frequency. Von Groll & Ewins [46] performed a harmonic balance method on a system where the stator had inertia as well as stiffness, which found both constant contact solutions but also some partial contact solutions. While this method was inefficient when initially locating such solutions, it was augmented with arc-length continuation to trace families of solutions once a solution had been found.

Cole & Keogh [47] emphasize the distinction between two forms of partial contact motion: *synchronous* partial that occurs at harmonics and subharmonics of the rotor shaft speed, and *asynchronous* partial contact where the frequency of impacts is apparently unrelated to the shaft speed. This work suggests that synchronous partial contact occurs when the rotor/stator system cannot be assumed to be axisymmetric, and this appears to be supported within the literature cited above. Solutions for asynchronous partial contact orbits are harder to solve, because the fundamental frequency of the asynchronous must be found in addition to the Fourier series components. In [47], this frequency is found by balancing the energy immediately before and after an impact accounting for the coefficient of restitution; hence relying on the assumption of instantaneous contact. The same authors use a similar assumption to solve synchronous responses in [48]. It is interesting to note the recent work of Mora *et al.* [49] who use recent techniques from piecewise-smooth dynamical systems theory to analyse the bifurcations that can occur at such impacts at primary resonances.

An intriguing new insight into asynchronous partial contact motion was demonstrated in the recent paper by Zilli *et al.* [50]. Here they find a condition for isolated bouncing motion that can coexist with the fundamental response curve, away from any primary resonances. They explained the onset of this motion through a synchronization condition between the forward and backward whirling modes of this system that made it susceptible to asynchronous partial contact motion. This led to an approximate prediction of the drive speeds that could cause this motion. In [51], the present authors reported a generalization of Zilli’s work to apply to any two modes of a two disc system, with any combination of forward and backward whirling. This showed further forms of asynchronous response, made possible by the increased number of linear whirling modes of the multi disc system. This condition is reproduced in §2 below and is argued here for the first time to be identifiable as form of internal resonance.

The main focus of the present work is to perform an in-depth brute-force numerical bifurcation analysis of a typical multiple degree-of-freedom rotating machine, with rotor stator contact. We find in many cases that multiple forms of asynchronous partial contact motion can occur at a single drive speed. We show how these can be related to underlying whirling modes of the system by the internal resonance condition. The ultimate goal is to gain an understanding of the conditions under which partial contact cycles might be triggered, and hence to understand how to avoid these potentially damaging oscillations in practice.

The rest of the paper is outlined as follows. Section 2 contains an explanation of different forms of vibration modes in a multiple degree-of-freedom rotating system and how these depend on drive speed. The internal resonance condition that predicts the onset of partial contact cycles in the presence of a stator is then derived, with care taken to explain the assumptions underlying the calculation. Section 3 then presents the example system on which we perform numerical computations, as well as outlining the numerical method used. Section 4 contains the results of a brute-force bifurcation analysis, together with a discussion of the origin of the different forms of partial contact solutions obtained. The results are presented for both a system with low contact stiffness and a purely elastic stator which permits a rich variety of responses, and then for a case more commonly encountered in practice, where the contacts have high stiffness and significant damping. It is shown that the internal resonance condition explains the onset of bouncing motion, even as more complex orbits evolve. Finally, §5 draws conclusions and suggests avenues for future work.

## 2. Analytical considerations

This section firstly presents a general mathematical description of the system under consideration, and how its motion may be described in terms of whirling modes of the underlying linear conservative system. It then shows how an internal resonance between these whirling modes is a necessary condition for asynchronous periodic contact.

### (a) General description of the system

We consider a general class of multiple degree-of-freedom systems that rotate about an shaft at a constant rotating speed *Ω*, subject to constant out-of-balance forces. We further suppose that the free rotor has light external damping, but can be subject to frictionless contact with a single concentric stator of fixed clearance, at a single point on the shaft. The shaft is assumed to be torsionally and axially rigid, hence these degrees of freedom are ignored in favour of purely lateral vibration modes. The shaft is assumed to have negligible internal damping, to avoid potential instabilities [1] that are not the focus of this work. Furthermore, it is assumed that all rotor bearings are isotropic and the stator clearance is circular with the same centre as the shaft. All elements of the system behave in a manner consistent with linear assumptions apart from possible rotor–stator contact; therefore, this is an example of a system with a single, local nonlinearity.

The equations of motion of this system can be written using the standard assumptions of Lagrangian mechanics as

2.1 $$\text{M}\ddot{\text{q}}+\Omega \text{G}\dot{\text{q}}+\text{K}\text{q}+\text{C}\dot{\text{q}}+\text{N}(\text{q},\dot{\text{q}})=\text{Re}\;(\Omega^{2}{\text{b}}_{0}\;e^{i\Omega t}),$$

where the vector $\text{q}\in R^{n}$ contains generalized rotations and displacements in a stationary coordinate frame. The matrices **M**, **C** and **K** are symmetric mass, damping and stiffness matrices respectively, and **G** is a skew-symmetric matrix of gyroscopic coupling terms. The vector **b**_0_ captures the distribution of out of balance forces which are written in complex form [1], and $i=\sqrt{-1}$. In addition, there is a nonlinear term $\text{N}(\text{q},\dot{\text{q}}),$ representing the rotor–stator contact which we discuss later.

This system may also be considered in a coordinate frame rotating with angular speed *Ω* about the shaft centre line. Hence a transformation exists between the two coordinate systems:

2.2 $$\text{q}=\text{T}(\Omega t)\tilde{\text{q}},$$

where **T**(*Ωt*) is a time-varying rotational transformation matrix, and $\tilde{\text{q}}$ represents generalized displacements in the rotating system. Under such a transformation, equation (2.1) may be transformed to the form

2.3 $$\text{M}\ddot{\tilde{\text{q}}}+\Omega \tilde{\text{G}}\dot{\tilde{\text{q}}}+\tilde{\text{K}}\tilde{\text{q}}+\text{C}\dot{\tilde{\text{q}}}+{\tilde{\text{K}}}_{c}\tilde{\text{q}}+\text{N}(\tilde{\text{q}},\dot{\tilde{\text{q}}})={\tilde{\text{b}}}_{0}.$$

The system matrices are assumed to be isotropic, i.e. invariant under axial rotation, hence **M** and **C** remain unchanged between equations (2.1) and (2.3). However, substitution of equation (2.2) into equation (2.1) requires expansion of derivatives using the chain rule, which results in additional terms which are incorporated into the rotational forms of the gyroscopic and stiffness matrices denoted $\tilde{\text{G}}$ and $\tilde{\text{K}}$, respectively. The term ${\tilde{\text{K}}}_{c}\tilde{\text{q}}$ contains forces that originate from the transformation of the term $\text{C}\dot{\text{q}}$ in equation (2.1); however, these are kept separate from $\tilde{\text{K}}\tilde{\text{q}}$ for convenience when separating the conservative parts of the system from the non-conservative parts. The matrix $\tilde{\text{K}}$ is symmetric, the matrices $\tilde{\text{G}}$ and ${\tilde{\text{K}}}_{c}$ are skew symmetric. Note that all matrices with a tilde are functions of shaft speed *Ω*. If it is assumed that the nonlinear force acts in a purely radial direction (as in frictionless contact) and is axisymmetric, the form of $\text{N}(\text{q},\dot{\text{q}})$ may be used in either coordinate system unchanged. Also note that the out of balance forcing term, has now become a constant forcing term instead of a harmonic time varying term. Specific details of these matrices and transformations are given later on.

### (b) Whirling modes of the underlying linear conservative system

If forcing, damping and nonlinearity are neglected, a linear conservative system is extracted from equation (2.3):

2.4 $$\text{M}\ddot{\tilde{\text{q}}}+\Omega \tilde{\text{G}}\dot{\tilde{\text{q}}}+\tilde{\text{K}}\tilde{\text{q}}=0.$$

Owing to the first derivative term in equation (2.4), it is convenient to transform it into state space form prior to solution:

2.5 $$\dot{\tilde{\text{y}}}=\tilde{\text{A}}\tilde{\text{y}},\;\tilde{\text{y}}=\left\{\begin{array}{c} \tilde{\text{q}}\\ \dot{\tilde{\text{q}}} \end{array}\right\}\;\mathrm{and}\;\tilde{\text{A}}=\left[\begin{array}{cc} \text{0} & \text{I}\\ {\text{M}}^{-1}\tilde{\text{K}} & \Omega {\text{M}}^{-1}\tilde{\text{G}} \end{array}\right].$$

Solutions to equation (2.5) have form

$$\tilde{\text{y}}={\tilde{\text{w}}}_{i}\;e^{{\tilde{s}}_{i}t},$$

where ${\tilde{\text{w}}}_{i}$ are the first-order eigenvectors and ${\tilde{s}}_{i}$ are the eigenvalues which are purely imaginary. From the definition of $\tilde{\text{y}}$, the first-order eigenvectors must have form $\tilde{\text{w}}={\left[{\tilde{\text{v}}}_{i},s_{i}{\text{v}}_{i}\right]}^{T}$, and, therefore, it is possible to extract the second-order eigenvectors that satisfy equation (2.4) directly with

$$\tilde{\text{q}}={\tilde{\text{v}}}_{i}\;e^{{\tilde{s}}_{i}t}.$$

If the number of degrees of freedom in equation (2.4) is *n*, there are 2*n* states in equation (2.5) and hence there are 2*n* eigenvalues and associated eigenvectors. The mode shapes are complex and appear in conjugate pairs, so that a motion based on a single conjugate pair of eigensolutions will have form

2.6 $$\tilde{\text{q}}=V_{i}{\tilde{\text{v}}}_{i}\;e^{{\tilde{s}}_{i}t}+V_{i}^{*}{\tilde{\text{v}}}_{i}^{*}\;e^{{\tilde{s}}_{i}^{*}t},$$

where an asterisk indicates complex conjugation and *V*_*i*_ is a complex amplitude. Evaluating equation (2.6) at any convenient point along the shaft will show a displacement that follows a circular path around the centre line, at an angular speed ${\tilde{\omega }}_{i}$, known as the whirl speed. If the motion is anticlockwise, the same direction as shaft speed *Ω*, the whirl speed ${\tilde{\omega }}_{i}$ is defined as positive and the motion is described as a forward whirl mode. Otherwise, the motion is described as a backward whirl mode and ${\tilde{\omega }}_{i}$ is negative. It may be shown that all eigenvalue pairs may be expressed as

$${\tilde{s}}_{i},{\tilde{s}}_{i}^{*}=\pm i{\tilde{\omega }}_{i}$$

and that all motions of the conservative system can be expressed as a superposition of multiple whirl modes in the form of equation (2.6).

A similar analysis could be performed starting with equation (2.1) instead of equation (2.3), hence deriving whirl modes in the stationary coordinate system. However, it can be seen from the form of the transformation between the two systems that whirl speeds in the stationary system will be related to those in the rotating system by

2.7 $$\omega_{i}={\tilde{\omega }}_{i}+\Omega$$

and that the whirl mode shapes will be identical. This relationship allows the possibility that a forward whirl mode in the stationary system is a backward whirl in the rotating system, if *Ω*>*ω*_*i*_. In the majority of rotordynamics literature, the greatest attention is paid to modes that represent forward whirl in the stationary frame, as these are excited by the out of balance forces and, therefore, determine linear critical rotation speeds. However, in this work all modes, either backward or forward, are equally relevant because impacts with the stator can excite any mode.

### (c) Internal resonance condition for asynchronous partial contact orbits

Section 2a established that the underlying linear system has responses in the forms of whirling motions, where the whirling speeds are functions of shaft speed *Ω*. These underlying modes will have a strong effect on the system response unless the nonlinear and non-conservative elements are strong, and therefore a partial contact motion must interact with these underlying modes to be sustained. Two limits in which such an observation can be made precise are when the stator nonlinearity is sufficiently weak, then a harmonic balance approximation can be made, as for example in [46]. Alternatively, in the rigid stator limit, the condition for the linear undamped system to have a periodic solution can be considered as the conditions for a grazing bifurcation (e.g. [49]). Further discussion on this point will be made in §5.

A response that predominately consists of motion in a single mode will consist of a circular orbit in an appropriate frame and hence will not be able to reproduce the complexity of an asynchronous partial contact orbit. Therefore, an analysis proceeds by considering *any* two whirling modes of the underlying linear system, with whirl speeds ${\tilde{\omega }}_{i}$ and ${\tilde{\omega }}_{j}$ in the rotating frame.

By definition, asynchronous partial contact is aperiodic with shaft speed *Ω* in the stationary coordinate system. Therefore, we consider motion in the rotating frame, as this means that the out of balance force is now a constant term and, therefore, does not directly affect the problem of seeking the fundamental period for oscillation. If it is assumed that the whirling speeds are be similar to those of the underlying linear conservative system, the condition for periodicity in the rotating system is that a rational ratio exists between the linear rotation speeds. This condition can be stated as

2.8 $$(p-q){\tilde{\omega }}_{i}=p{\tilde{\omega }}_{j},$$

where *p* and *q* are integers. Substituting equation (2.7) into equation (2.8) enables us to rewrite this relationship in terms of the stationary-frame whirl speeds:

2.9 $$q\Omega =p(\omega_{j}-\omega_{i})+q\omega_{i}.$$

The reason for the slightly contrived form of equation (2.8) becomes clear when equation (2.9) is compared with the work of Zilli *et al.* [50]. That paper studied the simplest form of the system described in Section 2a, with just 2 d.f., representing single forward and backward whirl modes (in the stationary frame). Let us call these modes ${\hat{\omega }}_{\mathrm{FW}}$ and ${\hat{\omega }}_{\mathrm{BW}}$, respectively. From a physical argument based on the periodic synchronization of the two whirling modes and the out of balance forcing phasor, a condition for partial contact orbits was given by Zilli *et al.* as

2.10 $$\hat{\Omega }=n({\hat{\omega }}_{\mathrm{FW}}+{\hat{\omega }}_{\mathrm{BW}})+{\hat{\omega }}_{\mathrm{FW}},$$

where the hats indicate the non-dimensional quantities used. Equation (2.10) is obviously identical to (2.9) under the substitution *q*=1, $\omega_{i}={\hat{\omega }}_{\mathrm{FW}}$ and $\omega_{j}=-{\hat{\omega }}_{\mathrm{BW}}$, noting that ${\hat{\omega }}_{\mathrm{BW}}$ was defined as being positive in [50], whereas the current work defines backward whirl as having negative whirl speed.

Note that for three or more whirling modes to participate in a periodic motion, all whirl speeds must be in a relationship in the form of equation (2.9) with all other whirl speeds, simultaneously. This is highly unlikely (and would occur for higher codimension) whereas for just two modes, the fact that whirl speeds vary with shaft speed means that equation (2.9) is a codimension-one condition. Note that for each *p* and *q*, as each modal frequency generally depends monotonically on *Ω*, there is likely to be at most a unique solution for each choice of whirling modes and each choice of integers *p* and *q*.

Understanding condition (2.8) as an internal resonance in the rotating coordinate system is simpler than the derivation of condition (2.9) in the stationary system, as performed in [50] or [51]. There, the argument considered the motion of phasors and the requirement for the phasor for each whirling mode and the applied shaft rotation to all be aligned. This ad hoc description masked the simpler formula (2.8) which can be understood as a straightforward internal resonance condition.

## 3. Example: a two disc rotor

We illustrate the predictive power of the above conditions on a specific example of the class of rotordynamic systems described in §2a. First, §2a gives details of the rotor system considered. Then §2b gives an example of how the insight of §2b can be applied to give initial predictions for where partial contact orbits may occur. Then §2c describes the brute force bifurcation methodology used to numerically investigate the response of the system.

### (a) Description and modelling

**Figure 1.**
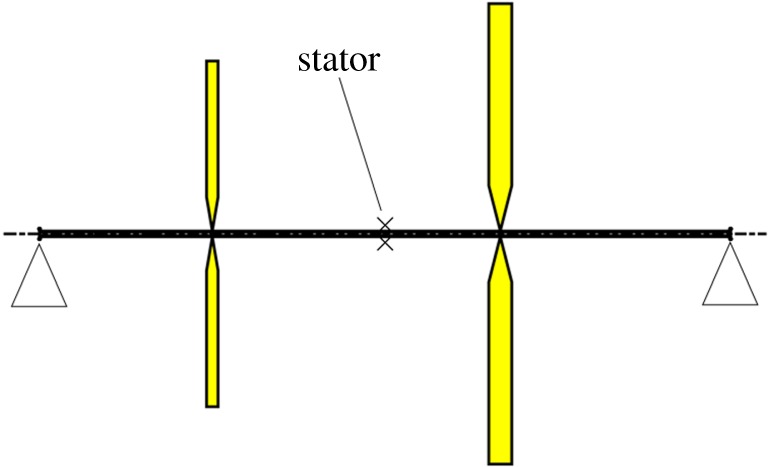
Test system. (Online version in colour.)

The system under consideration can be derived from the configuration shown in figure 1 which consists of a tube with two asymmetrically mounted, non-identical discs. The rotor is mounted on pinned bearings at each end of the shaft, with a stator in the form of a snubber ring, mounted at the centre of the shaft with a fixed clearance. The shaft is assumed to be constructed from a steel (*E*=210×10^9^ Pa, *ν*=0.3) tube with 5 mm outer diameter and thickness 1 mm. The discs are also made of steel with radii 0.15 m and 0.2 m and thicknesses 0.01 m and 0.02 m, respectively. The shaft is 0.60 m long, with the smaller disc located 0.15 m from the left-hand end, and the larger disc located 0.20 m from the right-hand end. The stator contact is modelled by assuming that the lateral vibrations of the shaft is subject to bilinear stiffness and damping. Specifically, the force exerted by the stator is assumed to be given by

3.1 $${\text{F}}_{c}=\left\{ \begin{array}{ll} -k_{s}(r-\delta )\left\{ \begin{array}{c} x_{c}\\ y_{c} \end{array}\}/\right.r-c_{s}\;\left\{\begin{array}{c} {\dot{x\;}\;}_{c}\\ {\dot{y\;}\;}_{c} \end{array}\right\}, & \text{if }r\geq \delta \\ \left\{\begin{array}{c} 0\\ 0 \end{array}\right\}, & \text{if }r<\delta , \end{array}\right.$$

where *k*_s_ is the stator contact stiffness, *c*_s_ the stator contact damping coefficient, *x*_c_ and *y*_c_ are the coordinates of the shaft centre taken in the plane of the stator, *δ* is the clearance of the stator and $r=\sqrt{x_{c}^{2}+y_{c}^{2}}$. In all cases presented here, *δ*=1×10^−3^ m.

Synchronous forcing is provided by a small off-centre eccentricity *ε* on the smaller disc. In order to ensure that initial transients decay within a reasonable time period to the steady state, light damping is applied along the length of the shaft. This is assumed to be external damping, to avoid potential instabilities caused by internal damping [1].

The configuration is initially modelled using the finite-element method. The mesh for this model is in the form of a shaft-line model, that is, all nodes are located along the centre line of the shaft. Thirteen nodes are spaced equally along the shaft, with the degrees of freedom at each node being vertical and lateral translation and rotation. Therefore, the model has *n*=48 d.f. where translation degrees of freedom have been eliminated from the end nodes by the assumption of pinned bearings. The shaft itself was represented by Timoshenko beam elements, and the discs by additional inertia terms at the nodes concerned. In all cases presented, the damping matrix was assumed proportional to the stiffness matrix, given by **C**=0.01 **K**. The methods in [1] were used to calculate all matrices in equation (2.1), through the free Matlab [52] scripts that accompany that work. Further details of the modelling process and the form of all the matrices derived are given in the appendix.

The model derived via this process we refer to as the ‘full model’. In what follows, simulations were carried out using a reduced *n*=8 d.f. reduced-order model. This is obtained from the full model by projecting onto the eigenvectors corresponding to the eight lowest-frequency modes of rotor for *Ω*=0 (which come in four pairs due to the symmetry in this case). Full details of the reduction process are also given in the appendix. All simulations were carried out at rotation speeds significantly below the frequency of the highest mode in the reduction. We ran several test cases, and we found no appreciable differences between the nature of the motion of the reduced model and the full model when projected back onto the first eight modes.

### (b) Predictions of potential internal resonance

**Figure 2.**
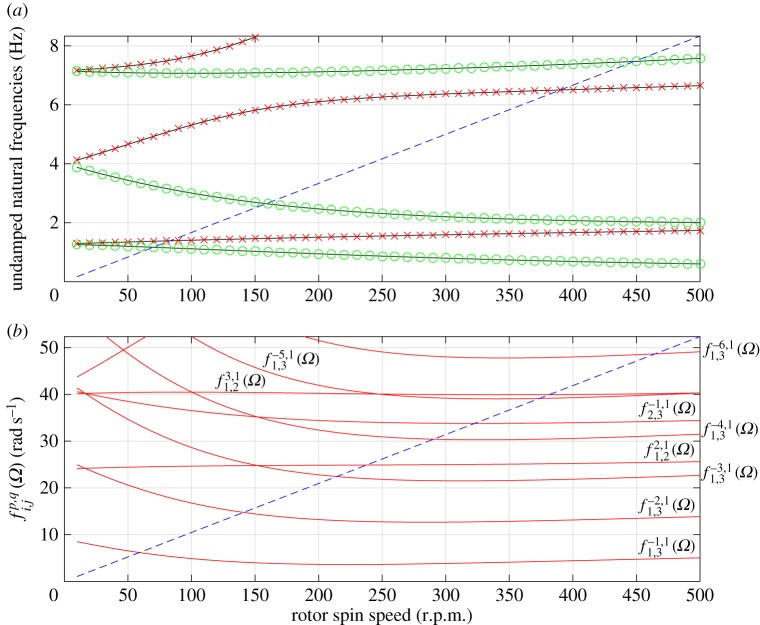
(*a*) Campbell diagram for test system, showing whirl frequencies in the stationary coordinate system. Crosses indicate forward whirls, circles indicate backward whirls. Dashed line indicates the shaft speed. (*b*) Evaluations of $f_{i.j}^{p,q}(\Omega )$ as given by equation (3.2). Labels indicate the choice of parameters used for the adjacent curve. Dashed line indicates the shaft speed. (Online version in colour.)

Figure 2*a* shows a Campbell diagram (linear modal frequencies *ω*_*j*_ versus shaft speed *Ω*) for the example system, where all frequencies are measured in the stationary coordinate system. This form of diagram is typically used to graphically predict the critical speeds at which a primary resonance occurs in a rotor system (e.g. [1]) by noting shaft speeds where a forward whirl frequency intersects the drive speed line. Note in the figure that we have distinguished between forward and backward whirls, although both forward and backward frequencies are represented as positive quantities, for convenience. One can, therefore, observe for this example that the primary shaft resonance would occur at about 80 r.p.m., whereas the second resonance does not occur until about 390 r.p.m.

A similar graphical method can be used to predict regions of potential asynchronous partial contact motion. First, we define a function *p* and *q*:

3.2 $$f_{i,j}^{p,q}(\Omega )=\frac{p}{q}[\omega_{j}(\Omega )-\omega_{i}(\Omega )]+\omega_{i}(\Omega ),$$

such that $f_{i,j}^{p,q}=\Omega$ for some integers *i*,*j*,*p* and *q* corresponds to the satisfaction of the internal resonance condition. Here *ω*_*i*_(*Ω*) and *ω*_*j*_(*Ω*) are calculated for each different shaft speed as described in §2b. The indices *i*,*j* can be any value from 1 to *n*, where the modes are ordered in terms of |*ω*|, which is the same order they appear from bottom to top of figure 2*a*.

The space of possible values of *p*,*q*,*i* and *j* is large, so ways of eliminating infeasible and duplicated cases are needed. First, note that changing the signs of *p* and *q* has no effect on the meaning of (2.9); hence we assume without loss of generality that *q* is positive (note that *q*=0 gives a trivial form for (2.8)). Furthermore, in simulations we found no appreciable regions of parameter space corresponding to motion resulting from resonances with *q*>1. This is presumably due to consequences of the theory of quasi-periodic motion (e.g. [53]) that the *Arnold tongues* associated with rotation numbers corresponding to higher *q* occupy narrower regions of parameter space and so are less likely to be excited in practice. Therefore, in what follows we only consider the case *q*=1. Another means of reducing the possible combinations is to note that if the choice of modes *i* and *j* are swapped in (3.2), then the same equation is obtained if we swap *p* with *q*−*p*. To eliminate this duplication, we assume without loss of generality in (3.2) that *j*<*i*. Finally, cases where *p*=1 or *p*=0 are also omitted, because these give trivial forms of equation (2.9).

Evaluations of equation (3.2) were performed for values of *p* from −10 to 10. The results of this sweep are shown in figure 2*b*; note that many choices of *p* result in curves that lie outside the region depicted. An intersection of a curve and the dashed line *ω*=*Ω* indicates that equation (2.9) is satisfied, and that therefore there is potential for internal resonance at that shaft speed.

It can be seen from figure 2*b* that there are numerous solutions to equation (2.9) in the region shown. However, this analysis has only considered the underlying linear whirl speeds; in practice, the detail of the mode shapes, damping, nonlinearity, non-conservative forces and initial conditions may all influence whether a partial contact orbit exists or is stable. More generally, in dynamical systems for which quasi-periodic motion is predicted by a linear or conservative analysis, it is known that internal resonances with sufficiently large Arnold tongues, in the presence of nonlinearity and damping, typically lead to appreciable parameter intervals in which there is stable phase-locked periodic motion. By contrast, those resonances with narrow Arnold tongues tend to not be excited (e.g. [53,54]). In our context, as nonlinearity only arises through contact, these phase-locked motions will necessarily be partial contact solutions. Owing to the nonlinear nature of these considerations, in practical situations we turn to numerical methods to see which of these potential internal resonances lead to observable partial contact motion.

### (c) Numerical bifurcation methodology

Bifurcation analysis is an invaluable framework for understanding nonlinear systems, by identifying and classifying points at which their responses undergo qualitative changes [54]. However, in cases such as this one with relatively little analytical knowledge of the underlying system and its responses, and with the further complication of a discontinuous nonlinearity, the simplest reliable means of obtaining a bifurcation diagram can be through conducting a large number of time-domain simulations. This approach, known as *brute force* bifurcation analysis, is adopted here.

At a given shaft speed, a randomized initial condition is created. A time simulation is then run for sufficient time for transients to decay. Simple properties are then used to quantify the final steady state motion—in this case, the motion is simply characterized by the maximum and minimum magnitude of displacement of the stator node during oscillation (note that a Poincaré analysis as used in [38] cannot be used because we have no natural choice of the sampling period). For these tests, the minimum and maximum are taken over 250 drive rotations once the data have settled.

The above procedure is repeated 25 times at each driving frequency; this effectively forms a *Monte Carlo* analysis of the stable steady state responses of the system at a given driving speed. A small step is then made in the shaft speed *Ω* and the process repeated, so that a complete one-parameter bifurcation diagram is obtained of solution response versus shaft speed.

To improve the performance of the simulation, the system was reduced to 8 d.f. (16 dimensions of phase space) by projection onto the first 8 linear undamped mode shapes of the non-rotating system. This includes all the major modes of the system; modes 9 and above are only linearly excited at much higher rotation speeds for for this system. The simulation itself was carried out in ODE45 in Matlab [52], with event detection used to locate changes between contacting and non-contacting motion. The event detection function halted the simulation every time the radial displacement of the contact node crossed the clearance threshold, and then the simulation would be restarted with optimal properties for the new state of contact or non-contact. Note that as part of this process, equation (3.1) requires that the radial displacement is evaluated at the contact node (which is a projection of linear modal vectors). Once contact has been established, the resulting force must also transformed back to the reduced modal form.

The random initial condition was generated as follows. The state space vector was set to ${\left\{\text{0}{\dot{\text{p}}}_{0}\right\}}^{T}$ where the reduced modal velocity vector ${\dot{\text{p}}}_{0}$ was populated with pseudo random numbers for each time simulation, hence the initial conditions were zero displacement with a random velocity. This vector was then scaled so that the non-dimensional initial kinetic energy given by $\frac{1}{2}{\dot{\text{p}}}_{0}^{T}{\dot{p}}_{0}$ was a constant value of 0.06 in each case.

## 4. Results

We present the results of the brute force bifurcation analysis for the example system, in two extreme cases. First, in §4a, the results for a relatively soft stator, with no damping are presented. Here we tend to find a wide range of excited internal resonances. Then, in §4b, a perhaps more physically realistic example (at least for laboratory implementation) is considered where the contact stiffness is high in relation to the shaft stiffness, and impacts are dissipative. While this appears to suppress many of the responses that occur in the previous case, partial contact orbits with rich dynamics are still seen to occur.

### (a) A low-stiffness snubber ring

We first present results where *k*_s_ has the relatively low value of 1×10^5^ N m^−1^ and no damping is applied in the stator i.e. *c*_s_=0. Therefore, this represents a snubber-ring type stator which does not totally suppress motion beyond *r*=*δ*. Eccentricity is introduced by the off-centre distance of disc 1 being *ϵ*=7.5×10^−4^ m.

**Figure 3.**
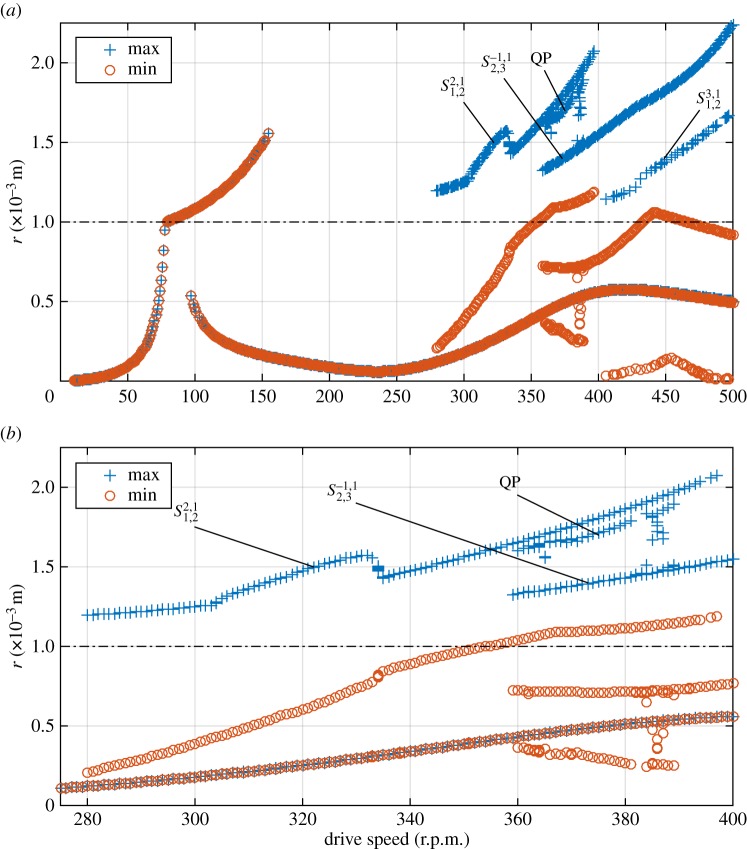
(*a*) Results of brute force bifurcation for *k*_s_=1×10^5^ N m^−1^. (*b*) Detail of region 280–400 r.p.m. Labels indicate the solution family that the response belong to. (Online version in colour.)

Figure 3 shows the results of the brute force bifurcation process as described in §3c. As can be seen, below 280 r.p.m. all markers coincide; as in all cases, the maximum and minimum *r* of the orbit are equal, it can be concluded that these cases always settle to synchronous whirling motion, where the radial displacement of the stator node remains constant. This motion is either linear forward (non-contacting) whirl, or in the case of the higher amplitude oscillations at 79–155 r.p.m., constant-contact forward whirl. In the region of approximately 100–150 r.p.m., both solutions are stable and the initial condition determines which orbit will occur in steady state. It can be seen that the linear forward whirl exists as a possible stable solution all the way up to 500 r.p.m.

**Figure 4.**
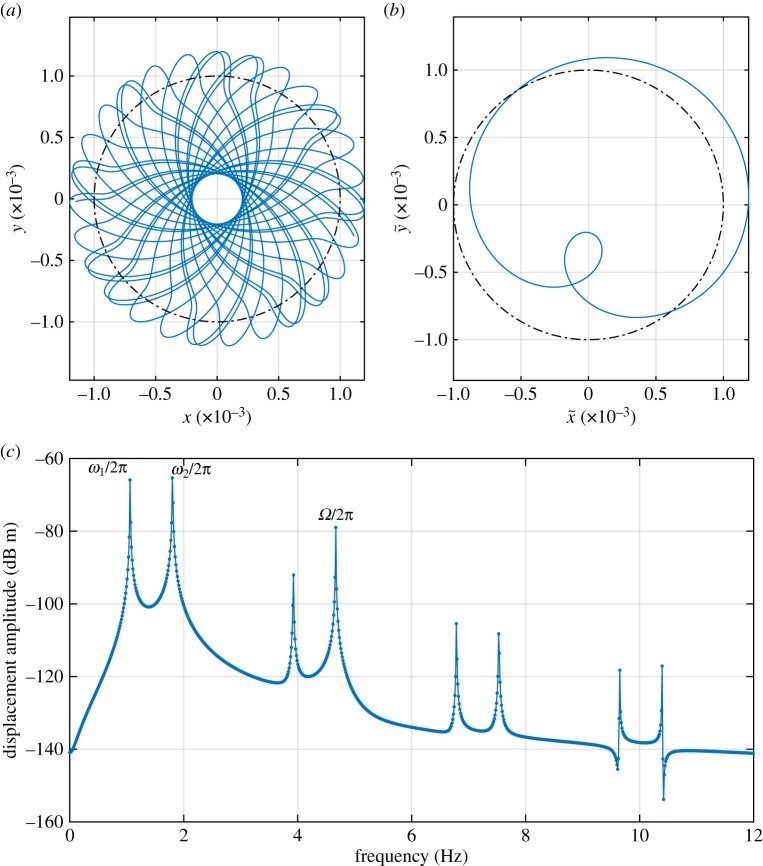
Steady-state partial contact orbit at drive speed 280 r.p.m. (*a*) Partial contact orbit in stationary coordinate system. Dotted-dashed line indicates the stator contact threshold. (*a*) Partial contact orbit in rotating coordinate system. Dotted-dashed line indicates the stator contact threshold. (*c*) FFT of lateral displacement in stationary coordinate system. (Online version in colour.)

At 280 r.p.m., a partial contact solution begins to occur, as shown by the presence of separate minimum and maximum markers in addition to those for the linear whirl case. The trace of the partial contact orbit for 280 r.p.m. is depicted in figure 4*a*, which shows that while this motion seems orderly, it is not periodic in stationary coordinates; the pattern shown will continually precess. However, figure 4*b* makes it clear that this motion is periodic and quite simple when viewed in a rotating coordinate system. In order to better understand this orbit, a fast Fourier transform (FFT) of the lateral displacement at the stator node in stationary coordinates is presented in figure 4*c*, which shows that the three largest peaks are at 1.06 Hz, 1.80 Hz and 4.66 Hz. The first two of these are slightly greater in magnitude than the first forward and backward whirling frequencies of the rotor as shown in figure 2*a*, and 4.66 Hz corresponds to the driving speed. If we take *ω*_1_=−1.06×2*π* rad s^−1^, and *ω*_2_=1.80×2*π* rad s^−1^, we find that these values approximately satisfy (2.9) for *p*=2. Hence this partial contact motion can be considered as an internal resonance between the first forward and backward whirls. The reason that the whirl speeds do not exactly match the linear predictions is believed to be that the nonlinearity induced by the stator has a stiffening effect typical of weakly nonlinear systems [54]. This also causes the onset of this motion to occur at the higher shaft speed of 280 r.p.m. compared with the 240 r.p.m. predicted by figure 2*b*. Accurate modelling of this stiffening effect is left to future work. All other peaks in figure 4 are much smaller in amplitude and can be shown to be the effect of harmonics of the fundamental rotating coordinate system frequencies when translated into the stationary frame.

In fact, the ability of nonlinearity to alter the whirling frequencies has a more profound effect, in allowing this form of solution to exist over a wide range of frequencies. It can be seen from figure 3 that the partial contact orbit at 280 r.p.m. is part of a family of solutions, that can be traced by following the line of maxima and corresponding minima. At all points on this line, analysis of the orbits similar to that given above reveals them to consist of the first two whirl modes, coupled with *p*=2. Therefore, this entire family is solutions is given a label in figure 3 of $S_{1,2}^{2,1}$, where the subscripts and superscripts reflect the underlying whirling modes that constitute the response, and the integers *p* and *q*, in the same was as used in equation (3.2). Note that by evaluating equation (2.8) with the given values, it may be seen that these responses are 2:1 internal resonances in the rotating coordinate system.

**Figure 5.**
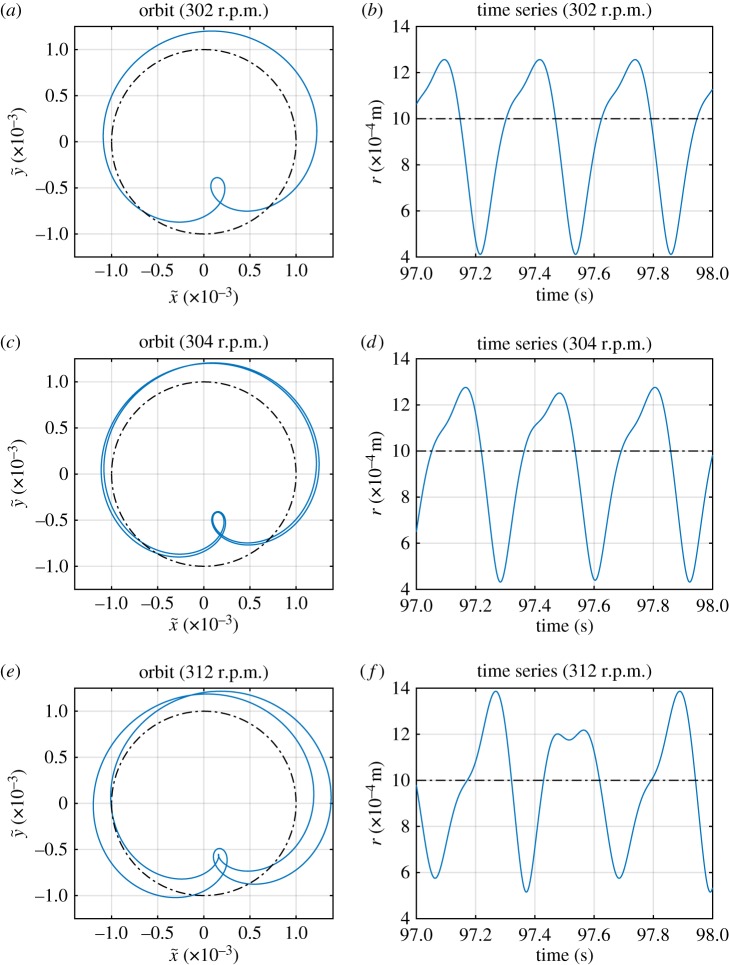
Detailed results from figure 3, solution $S_{1,2}^{2,1}$. (*a*,*c*,*e*) Orbits in rotating coordinate system at 302 r.p.m., 304 r.p.m. and 312 r.p.m., respectively. (*b*,*d*,*e*) Excerpt of the time series of *r* at 302 r.p.m., 304 r.p.m. and 312 r.p.m., respectively. Dotted-dashed line indicates the stator contact threshold. (Online version in colour.)

By following the lines of maxima and minima in figure 3, it may be seen that solution family $S_{1,2}^{2,1}$ extends up to 397 r.p.m. However, it may be seen that there are discontinuities in the amplitudes of responses as the drive speed increases. It emerges that these correspond to change-of-period bifurcations, as seen in figure 5, which shows how the ‘kink’ in the line of maxima at 303 r.p.m. corresponds to a period-doubling. This shows how at 302 r.p.m. all contacts are identical, whereas at 303 r.p.m. there is a gentle oscillation in the maximum *r* that is reached. By 312 r.p.m. this has grown into alternate contacts being very different to each other. Thus, it can be seen that substantially different responses can occur, but composed of the same form of synchronization of the underlying linear modes.

The other families of responses that are labelled in figure 3 were identified using a similar process to above. By evaluation of equation (2.8), it may be seen that the family $S_{2,3}^{-1,1}$ gives another 2:1 internal resonance in the rotating system, but this time between modes 2 and 3, and that $S_{1,2}^{3,1}$ gives a 3:2 resonance between the first two modes.

**Figure 6.**
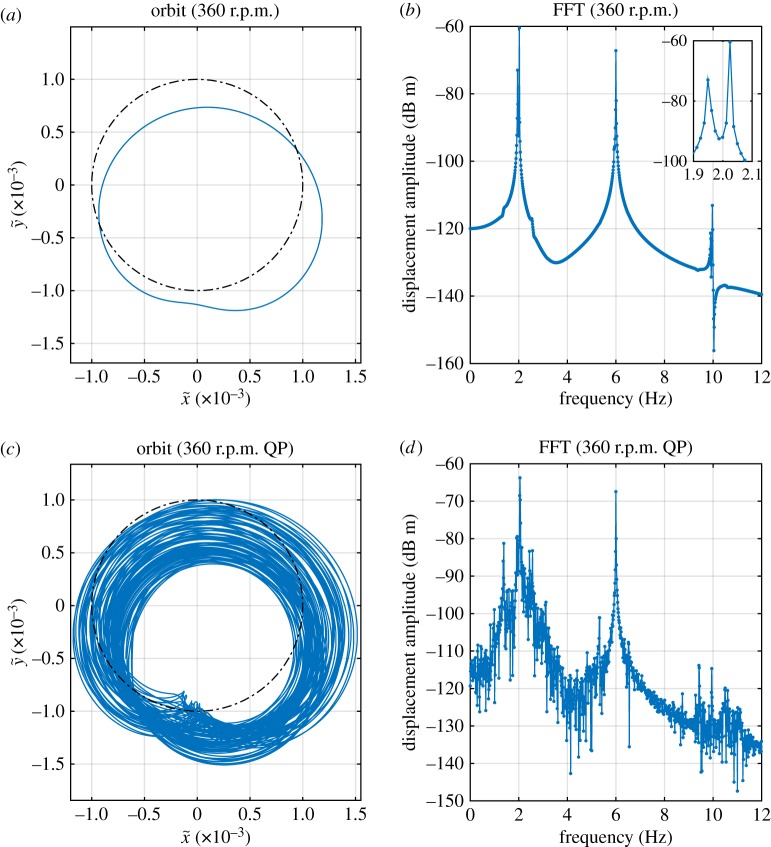
Detailed results from figure 3, 360 r.p.m. (*a*) Orbit of $S_{2,3}^{-1,1}$ solution in rotating coordinates. (*b*) FFT of lateral displacement in stationary coordinates of $S_{2,3}^{-1,1}$ solution (note there are two closely spaced peaks, at 1.95 Hz and 2.03 Hz). (*c*) Orbit of *QP* solution in rotating coordinates. (*d*) FFT of *QP* solution. (Online version in colour.)

The exception to this is the solution family labelled ‘QP’. This appears to be a quasi-periodic form of the family $S_{2,3}^{-1,1}$. This may be seen in figure 6, where figure 6*b* shows peaks at 1.95 Hz and 2.03 Hz, denoting the whirling mode contributions, and at 6.00 Hz denoting the synchronous forcing. Figure 6*d* shows a similar picture, but with significant additional broadband noise, a sign of chaotic motion. Another view of these orbits shown in figure 6*a*,*c*. While the two orbits show some similarity to one another, the ‘QP’ orbit in figure 6*c* clearly shows a bounded chaotic response.

The final feature of figure 3 that should be mentioned is the small number of ‘stray’ points that do not appear to be part of any solution family at 387 r.p.m. These points might be considered to be false readings, caused by the fact that at this driving speed many runs took an exceptionally long time to reach a steady state, and had not done so by the time that the response was sampled. This seemed to be because this particular drive speed is near the end of the region of stability for the QP response; the time series appear to be similar to the quasi-periodic orbit for a long time, before settling down to one of the periodic responses.

### (b) A rigid, damped stator

In many cases, the stator will be highly rigid compared with the shaft. Furthermore, the stator will have an additional damping effect, and this will act to suppress many of the oscillations that make large incursions into the contact region as seen in figure 3.

**Figure 7.**
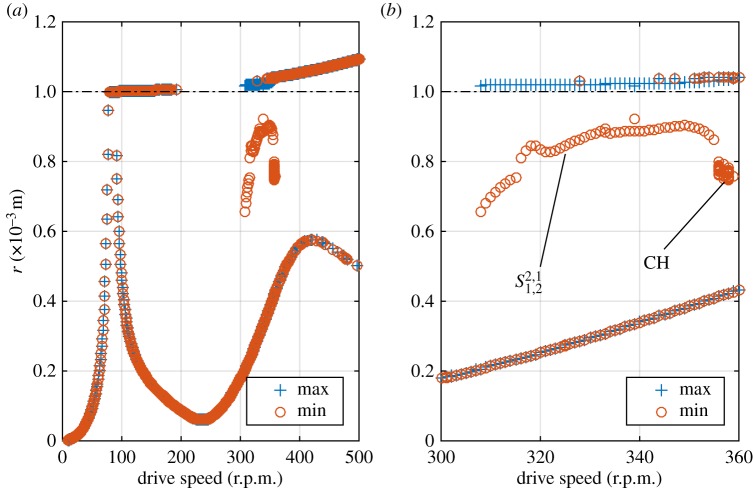
(*a*) Results of brute force bifurcation for *k*_s_=1×10^7^ N m^−1^ with contact damper *c*_s_=3.163×10^4^ N s m^−1^. (*b*) Detail of region 300–360 r.p.m. Labels indicate solution family, dotted-dashed line indicated clearance contact threshold. (Online version in colour.)

In order to see these effects, a second brute force diagram is presented with *k*_s_=1×10^7^ N m^−1^ and also giving the contact model a damper of *c*_s_=1.581×10^4^ Ns m^−1^. The results of this are shown in figure 7. It may be seen that this rotor features a second region of constant contact response beginning at approximately *Ω*=350 r.p.m.; however, as the focus of this work is partial contact response this is not discussed further. It is clear that the stiffer but less elastic contacts have had the effect of causing the majority of stable partial contact orbit solutions to disappear.

**Figure 8.**
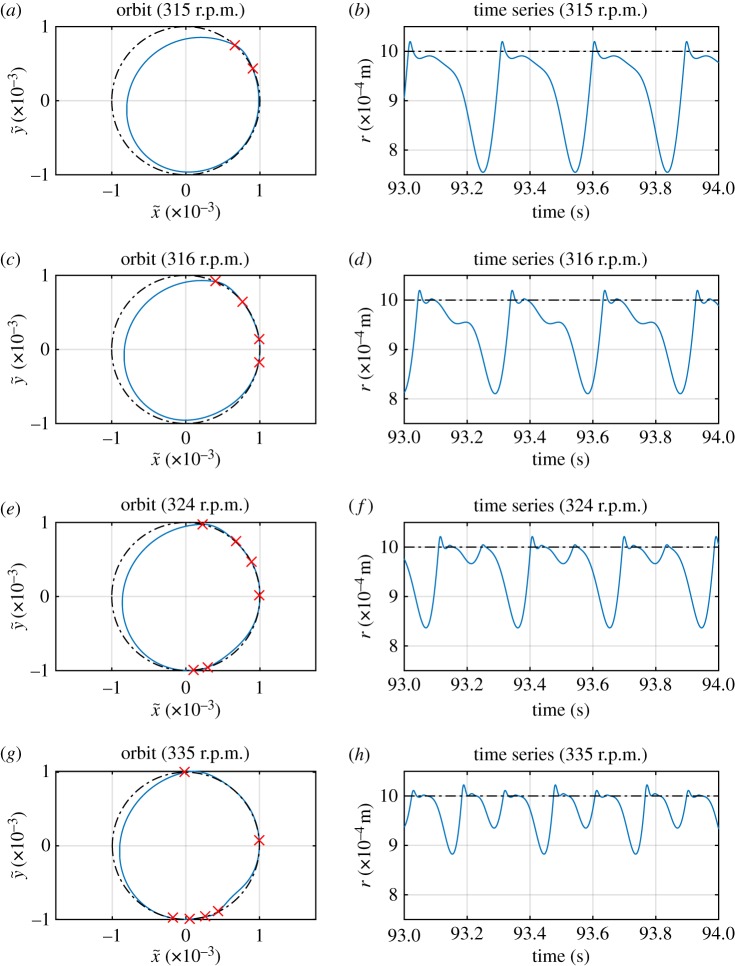
Time series and orbits of partial contact solutions from family $S_{1,2}^{2,1}$ in figure 7, at various drive speeds. Dotted-dashed lines show the stator contact threshold. The crosses in (*a*,*c*,*e*,*g*) indicate where contact with the stator is established or broken. (Online version in colour.)

**Figure 9.**
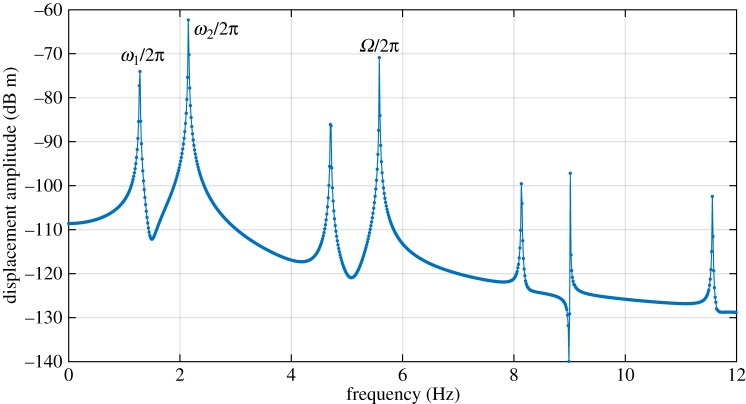
Frequency spectrum of lateral response in stationary coordinates for rotor with *k*_s_=1×10^7^ N m^−1^ with contact damper *c*_s_= 3.163×10^4^ N s m^−1^, driven at 335 r.p.m. and exhibiting $S_{1,2}^{2,1}$ response. (Online version in colour.)

The remaining partial contact responses all derive from the same solution family $S_{1,2}^{2,1}$, although now it does not occur until a shaft speed of 308 r.p.m. is reached, highlighting that the predictions of figure 2 become increasingly approximate as the harshness of the nonlinearity is increased. However, it may be noted that in this case the way that the response evolves as the driving speed increases is different to the previous case. Figure 8 shows a series of time signals of *r*(*t*), with increasing rotor speeds. The period-doubling seen earlier does not occur; however, the orbits start to contain multiple impacts in each cycle (this may be seen by observing the crosses that signify crossings of the clearance threshold). However, despite the increasing complexity of the motion, the two underlying whirling speeds (and the forcing frequency) remain clearly visible in the spectrum of displacement as shown in figure 9. This case also demonstrates that the impact frequency cannot always be treated as the fundamental frequency of the orbit for this system.

**Figure 10.**
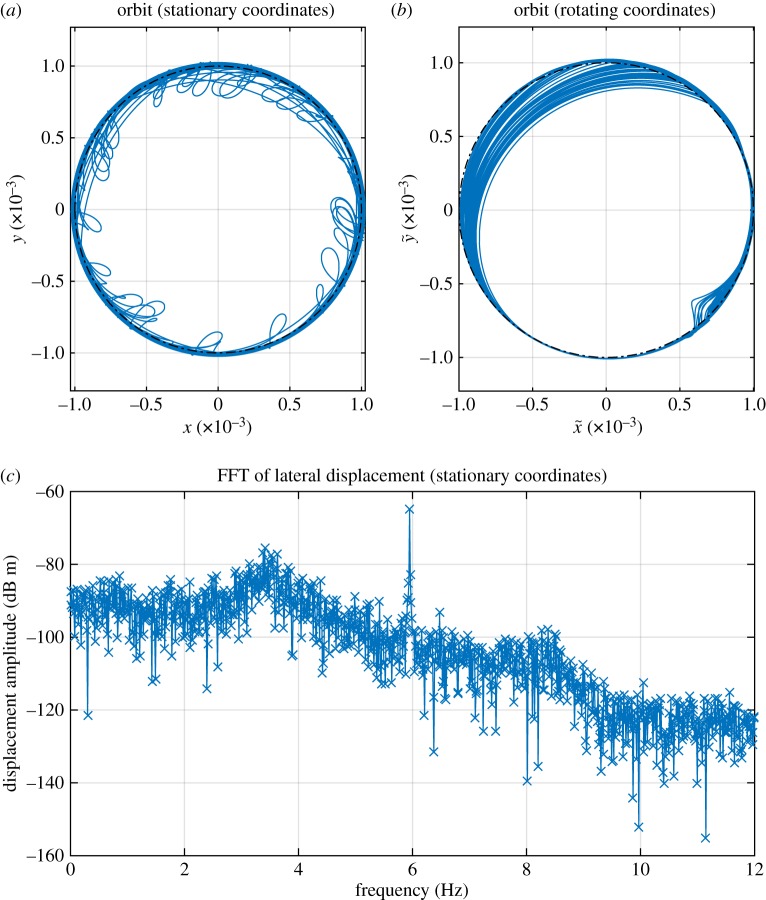
Chaotic orbit for *k*_s_=1×10^7^ N m^−1^ with contact damper *c*_s_=3.163×10^4^ N s m^−1^, at 357 r.p.m. (Online version in colour.)

The final region of particular interest is the solution family labelled *CH*, where figure 7 shows a large number of points with slightly different minimum values for *r*. The reason for this is the onset of chaotic motion, as the period of oscillation is now so long that even the long time period used for the simulation is insufficient to establish a consistent minimum. An example of the chaotic orbit in stationary coordinates is shown in figure 10*a*. However, when viewed in a rotating coordinate system, as shown in figure 10*b*, the motion is revealed to still be ordered and bounded. Unfortunately, figure 10*c* shows that the displacement spectrum now shows so much noise due to chaos that it is not possible to categorize this orbit in terms of an underlying internal resonance condition, with the only distinct frequency component being at the drive frequency.

## 5. Conclusion

This work has provided for the first time a coherent theory for the onset of bouncing motion, which we have termed asynchronous partial contact orbits, in a multi degree-of-freedom rotor–stator systems with contacts. Such motions have been observed in practice in a number of situations, not least in turbomachinery, drillstrings, driveshafts, etc. The surprising nature of these motions is that they appear to occur well away from any primary rotor resonances. Moreover, they tend to coexist with free, non-resonant, non-contacting whirl modes. The implication in practice is that transient effects can push the rotor from (desirable) non-contacting whirl, into these more violent, potentially damaging bouncing motions. Rather than trying to seek the nature of the transient that can push the dynamics into these undesirable states, our focus here has been to come up with a rational criterion for deciding whether these bouncing orbits are possible at all.

In particular, we have argued that the onset of these motions can be predicted by an internal resonance condition that links any two of the system’s linear whirl frequencies. The fact that whirling mode speeds are a function of the shaft speed means that certain shafts speeds become particularly susceptible to the onset of these motions. This interpretation can also be shown to be equivalent to a phasor synchronization condition that was discussed in previous works [50,51]. By performing a simple linearized calculation of the dependence of the whirling frequencies on drive speed, we can come up with precise predictions of the drive speeds at which these modes might become excited.

The internal resonance condition we have uncovered can only be considered as a necessary, rather than sufficient, condition for the onset of bouncing periodic motion. It is not clear *a priori* whether or not the corresponding resonant motion will be excited by the presence rotor–stator contact. We have illustrated this by looking at two very different limit cases of the nature of the contact for a typical rotordynamic system with a high number of degrees of freedom. The two limiting cases represent a compliant snubber ring, and a rigid, impacting constraint. The latter case is found to excite fewer resonances than the former. Another weakness is that the nonlinearity seems to shift the drive speed value at which the resonance is first seen; in all cases, the observed drive speed seems to be slightly higher than predicted. This may be because of the basin of attraction of the bouncing solution being vanishingly small at its point of onset. It may also be because of the effective stiffening caused by the nonlinearity. Finally, and most crucially, the internal resonance criterion does not predict the range of *Ω*-values over which a particular class of bouncing orbit may exist, nor whether period-doubling or other routes to chaos may occur at the high-*Ω* limit.

All of these more detailed questions require further nonlinear analysis. This is left to future work. It is interesting to observe though that all orbits appear to bifurcate from their onset point in the direction of increasing drive speed *Ω*. To establish this, and to gain insight into how the amplitude and characteristics of the bouncing orbits evolve as *Ω* is increased we have performed preliminary calculations using two different methods. The first involves harmonic balance, which applies best in the case of a soft snubber-ring-type constraint. The second involves a rigid impact analysis, using the *discontinuity mapping* methodology from the theory of piecewise-smooth dynamical systems (e.g. [55]). The results appear promising, but will be presented in detail elsewhere. Finally, experimental verification of the theory presented here is pressing, another avenue we are actively pursuing.

## Figures and Tables

**Figure 11.**
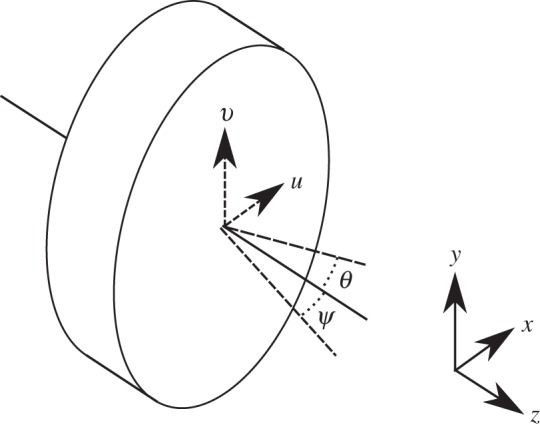
Coordinate system.

## Appendix A. Details of matrix derivations

**(a) System matrices**

The system is modelled following [1], by adapting the Matlab [52] scripts that accompany this work. The system is a shaft line model where each node *k* has 4 d.f.:

A 1 $${\text{q}}_{k}={\left[u_{k},v_{k},\theta_{k},\psi_{k}\right]}^{T},$$

which are illustrated in figure 11. The shaft consists of two-node beam elements, hence each element has degrees of freedom:

A 2 $${\text{q}}_{e}={\left[u_{k},v_{k},\theta_{k},\psi_{k},u_{k+1},v_{k+1},\theta_{k+1},\psi_{k+1}\right]}^{T}.$$

The shaft is assumed to be a Timoshenko beam neglecting rotary inertia hence the element stiffness matrix is

A 3 $${\text{K}}_{e}=\frac{EI}{(1+\phi )L^{3}}\left[\begin{array}{cccccccc} 12 & 0 & 0 & 6L & -12 & 0 & 0 & 6L\\ 0 & 12 & -6L & 0 & 0 & -12 & -6L & 0\\ 0 & -6L & (4+\phi )L^{2} & 0 & 0 & 6L & (2-\phi )L^{2} & 0\\ 6L & 0 & 0 & (4+\phi )L2 & -6L & 0 & 0 & (2-\phi )L^{2}\\ -12 & 0 & 0 & -6L & 12 & 0 & 0 & -6L\\ 0 & -12 & 6L & 0 & 0 & 12 & 6L & 0\\ 0 & -6L & (2-\phi )L^{2} & 0 & 0 & 6L & (4+\phi )L^{2} & 0\\ 6L & 0 & 0 & (2-\phi )L^{2} & -6L & 0 & 0 & (4+\phi )L^{2} \end{array}\right],$$

where *ϕ*=12*EI*/*GκAL*^2^ and symbols have their usual meanings, and the mass matrix is

A 4 $${\text{M}}_{e}=\frac{\rho AL}{840{\left(1+\phi \right)}^{2}}\left[\begin{array}{cccccccc} m_{1} & 0 & 0 & m_{2} & m_{3} & 0 & 0 & m_{4}\\ 0 & m_{1} & -m_{2} & 0 & 0 & m_{3} & -m_{4} & 0\\ 0 & -m_{2} & m_{5} & 0 & 0 & m_{4} & m_{6} & 0\\ m_{2} & 0 & 0 & m_{5} & -m_{4} & 0 & 0 & m_{6}\\ m_{3} & 0 & 0 & -m_{4} & m_{1} & 0 & 0 & -m_{2}\\ 0 & m_{3} & m_{4} & 0 & 0 & m_{1} & m_{2} & 0\\ 0 & -m_{4} & m_{6} & 0 & 0 & m_{2} & m_{5} & 0\\ m_{4} & 0 & 0 & m_{6} & -m_{2} & 0 & 0 & m_{5} \end{array}\right],$$

where

$$\begin{array}{rl} m_{1} & =312+588\phi +280\phi^{2}\\ m_{2} & =(44+77\phi +35\phi^{2})L\\ m_{3} & =108+252\phi +140\phi^{2}\\ m_{4} & =-(26+63\phi +35\phi^{2})L\\ m_{5} & =(8+14\phi +7\phi \phi )L^{2}\\ \;\mathrm{and}\;m_{6} & =-(6+14\phi +7\phi^{2})L^{2}. \end{array}$$

The gyroscopic matrix for each element is

A 5 $${\text{G}}_{e}=\frac{-I_{p}}{15L{\left(1+\phi \right)}^{2}}\left[\begin{array}{cccccccc} 0 & -g_{1} & g_{2} & 0 & 0 & g_{1} & g_{2} & 0\\ g_{1} & 0 & 0 & g_{2} & -g_{1} & 0 & 0 & g_{2}\\ -g_{2} & 0 & 0 & -g_{3} & g_{2} & 0 & 0 & -g_{4}\\ 0 & -g_{2} & g_{3} & 0 & 0 & g_{2} & g_{4} & 0\\ 0 & g_{1} & -g_{2} & 0 & 0 & -g_{1} & -g_{2} & 0\\ -g_{1} & 0 & 0 & -g_{2} & g_{1} & 0 & 0 & -g_{2}\\ -g_{2} & 0 & 0 & -g_{4} & g_{2} & 0 & 0 & -g_{3}\\ 0 & -g_{2} & g_{4} & 0 & 0 & g_{2} & g_{3} & 0 \end{array}\right],$$

where

$$\begin{array}{rl} g_{1} & =36\\ g_{2} & =(3-15\phi )L\\ g_{3} & =(4+5\phi +10\phi^{2})L^{2}\\ \;\mathrm{and}\;g_{4} & =(-1-5\phi +5\phi^{2})L^{2}. \end{array}$$

Equations (A 3)–(A 5) are combined in the usual way to obtain **K**, **M** and **G**. Furthermore, for nodes where discs are located, an addition to the mass matrix is made

A 6 $${\text{M}}_{d}\;=\left[\begin{array}{cccc} M_{d} & 0 & 0 & 0\\ 0 & M_{d} & 0 & 0\\ 0 & 0 & I_{d} & 0\\ 0 & 0 & 0 & I_{d} \end{array}\right]$$

at the appropriate degrees of freedom, and an addition to the gyroscopic matrix is also made

A 7 $${\text{G}}_{d}=\left[\begin{array}{cccc} 0 & 0 & 0 & 0\\ 0 & 0 & 0 & 0\\ 0 & 0 & 0 & I_{p}\\ 0 & 0 & -I_{p} & 0 \end{array}\right]$$

In equations (A 6) and (A 7), *M*_*d*_ is the mass of the disc, *I*_*d*_ is the disc’s moment of inertia about the lateral axes and *I*_*p*_ is its moment of inertia about the *z*-axis. The assumption of simple pinned bearings means that these can be implemented simply by removing the relevant translational degrees of freedom from all matrices. The damping matrix is assumed proportional to the stiffness matrix, hence **C**=0.01**K**.

The system is assumed to have an off-centre displacement on the smaller disc equal to *ϵ*=0.75×10^−3^m. Hence forcing vector **b**_0_ is given as

A 8 $${\text{b}}_{0}=\epsilon M_{d1}{\left[0\;\cdots \;0\;1\;-i\;0\cdots 0\right]}^{T},$$

where *M*_*d*1_ is the mass of the disc concerned, and the two non-zero terms occur at the degrees of freedom relating to displacement of the smaller disc.

For this system, given the arrangement of the degrees of freedom, the transformation matrix from rotating coordinates **T** used in (2.2) is a block diagonal matrix, where each block is the following:

A 9 $${\text{T}}_{k}(\Omega t)=\left[\begin{array}{cc} \cos\Omega t & -\sin\Omega t\\ \sin\Omega t & \cos\Omega t \end{array}\right],$$

which when substituted into the equation of motion gives

A 10 $$\begin{array}{rl} \tilde{\text{G}} & =\text{G}+2\text{M}\text{J}, \end{array}$$

A 11 $$\begin{array}{rl} \tilde{\text{K}} & =\text{K}-\Omega^{2}\text{M}+\Omega^{2}\text{G}\text{J} \end{array}$$

A 12 $$\begin{array}{rl} \;\text{and}\;{\tilde{\text{K}}}_{c} & =\Omega \text{C}\text{J}, \end{array}$$

where **J** is a block diagonal matrix where the block elements are $\left[\begin{array}{cc} 0 & -1\\ 1 & 0 \end{array}\right]$ i.e.

A 13 $$\text{J}=\left[\begin{array}{ccccc} 0 & -1 & 0 & 0 & \cdots \\ 1 & 0 & 0 & 0 & \\ 0 & 0 & 0 & -1 & \\ 0 & 0 & 1 & 0 & \\ \vdots & & & & \ddots \end{array}\right].$$

**(b) Reduced matrices for numerical simulation**

In order to perform numerical simulation, as part of the brute force bifurcation method in §3c, the system is reduced using non rotating modes. The transformed variable **p** is related to **q** by

A 14 q=Φp,

where ***Φ*** is formed by the first 8 mass normalized eigenvectors of **M**^−1^**K**; note that these will have four separate eigenvalues, each repeated twice due to symmetry between *x* and *y* directions of the structure. Hence transforming equation (2.1) gives

A 15 $$\ddot{\text{p}}+\Omega \Phi^{T}\text{G}\Phi \dot{\text{p}}+\Phi^{T}\text{K}\Phi \text{p}+\Phi^{T}\text{C}\Phi \dot{\text{p}}+\Phi^{T}\text{N}(\Phi \text{p},\Phi \dot{\text{p}})=\Phi^{T}ℜ(\Omega^{2}{\text{b}}_{0}\;e^{i\Omega t}).$$

The values of the reduced matrices are as follows:

A 16 $$\begin{array}{rl} \Phi^{T}\text{G}\Phi & =\left[\begin{array}{cccccccc} 0 & -0.1791 & 0 & -0.4710 & -0.2132 & 0 & 0 & -0.2400\\ 0.1791 & 0 & 0.4710 & 0 & 0 & 0.2132 & -0.2400 & 0\\ 0 & -0.4710 & 0 & -1.5056 & -0.7150 & 0 & 0 & 0.0299\\ 0.4710 & 0 & 1.5056 & 0 & 0 & 0.7150 & 0.0299 & 0\\ 0.2132 & 0 & 0.7150 & 0 & 0 & 0.3430 & 0.0966 & 0\\ 0 & -0.2132 & 0 & -0.7150 & -0.3430 & 0 & 0 & 0.0966\\ 0 & 0.2400 & 0 & -0.0299 & -0.0966 & 0 & 0 & 1.9611\\ 0.2400 & 0 & -0.0299 & 0 & 0 & -0.0966 & -1.9611 & 0 \end{array}\right], \end{array}$$

A 17 $$\Phi^{T}\text{K}\Phi =\mathrm{diag}([64.26\;64.26\;630.96\;630.96\;2022.08\;2022.08\;6646.04\;646.04])$$

A 18 $$\Phi^{T}\text{C}\Phi =\mathrm{diag}([0.643\;0.643\;6.310\;6.310\;20.221\;20.221\;66.460\;66.460]),$$

where diag([⋯ ]) gives a diagonal matrix where the diagonal terms are the elements of the row vector.

Noting that only the translational degrees of freedom at the stator node participate in the nonlinearity defined in equation (3.1), the nonlinear term in equation (A 15) can be reduced as follows:

A 19 $$\Phi^{\text{T}}\text{N(}\Phi \text{p},\Phi \dot{\text{p}})=\Phi_{\text{c}}^{T}{\text{F}}_{c}(\Phi_{\text{c}}p,\Phi_{\text{c}}\dot{\text{p}}),$$

where ***Φ***_c_ consists of the two rows of ***Φ*** relating to the translational displacements (*u* and *v*) at the stator node:

A 20 $$\Phi_{C}=\left[\begin{array}{cccccccc} 0 & -\text{0.2237} & 0 & -\text{0.0678} & \text{0.0654} & 0 & 0 & -\text{0.0996}\\ -\text{0.2237} & 0 & -\text{0.0678} & 0 & 0 & \text{0.0654} & \text{0.0996} & 0 \end{array}\right].$$

This matrix is also used when projecting the results of simulation back to the displacements at the stator node, as is done in all results figures.

In a similar manner to the reduction of the nonlinear term, the forcing term can be reduced by noting that it only affects translational degrees of freedom at the node of the smaller disc. Hence,

A 21 $$\Phi^{T}\text{Re}(\Omega^{2}{\text{b}}_{0}\;e^{i\Omega t})=\Phi_{f}^{T}\text{Re}(\Omega^{2}{\text{b}}_{f}\;e^{i\Omega t}),$$

where **b**_f_ consists of the two non-zero elements of **b**_0_ and ***Φ***_f_ consists of the two rows of ***Φ*** relating to the forced degrees of freedom:

A 22 $$\Phi_{f}=\left[\begin{array}{cccccccc} 0 & -0.1512 & 0 & -0.1295 & 0.3739 & 0 & 0 & 0.0351\\ -0.1512 & 0 & -0.1295 & 0 & 0 & 0.3739 & -0.0351 & 0 \end{array}\right].$$
